# Feasibility and postoperative analgesic profile of multimodal analgesia including combined serratus anterior plane block in minimally invasive cardiac surgery: a prospective observational study

**DOI:** 10.1186/s13019-026-04193-8

**Published:** 2026-04-30

**Authors:** Emine Nilgün Zengin, Aslıhan Aykut, Ayşegül Özgök, Musa Zengin, Onur Küçük, Asena İrem Yıldız, Fatih Üngör, Ali Alagöz, Nevriye Salman, Zeliha Aslı Demir

**Affiliations:** 1 Department of Anesthesiology and Reanimation, University of Health Sciences, Ankara Bilkent City Hospital, Ankara, Turkey; 2 Department of Anesthesiology and Reanimation, University of Health Sciences, Ankara Etlik City Hospital, Ankara, Turkey; 3 Department of Anesthesiology and Reanimation, University of Dokuz Eylül, School of Medicine, İzmir, Turkey; 4 Department of Anesthesiology and Reanimation, University of Health Sciences, Ankara Atatürk Sanatorium Training and Research Hospital, Ankara, Turkey; 5 Üniversiteler Mahallesi, Rıfat Börekçi Caddesi, No: 9. Ankara Bilkent City Hospital, Department of Anesthesiology and Reanimation, Ankara, Turkey

**Keywords:** Acute pain, Combination, Minimally invasive cardiac surgery, Plane blocks, Serratus anterior plane block

## Abstract

**Background:**

Although minimally invasive cardiac surgery (MICS) is less invasive, patients may still experience significant postoperative pain. This observational study aimed to evaluate the feasibility and postoperative analgesic profile associated with the combined serratus anterior plane block (CSAPB), a component of multimodal analgesia in patients undergoing MICS.

**Methods:**

Twenty patients, aged 18 to 80, who underwent MICS and provided written informed consent, were included in the study. Standard cardiac anesthesia monitoring was performed. Ultrasound-guided CSAPB was performed preemptively after induction of general anesthesia as part of a multimodal analgesia strategy, using a total of 40 mL of 0.25% bupivacaine, with 20 mL administered into each of the deep and superficial serratus anterior planes. All patients received intravenous acetaminophen 1 g and tramadol 1 mg·kg^− 1^ at the end of surgery. Within the first 24 h, patients received 1 gram of acetaminophen every 6 h. Visual Analog Scale (VAS) scores were recorded at 0, 2, 4, 8, and 12 h after extubation. If the VAS score exceeded 40 mm at any point, the initial treatment was tramadol at a dosage of 1 mg·kg^− 1^. If pain continued, 0.5 mg·kg^− 1^ morphine was planned.

**Results:**

The hemodynamic parameters remained within clinically acceptable ranges. In 2 patients (10%), VAS scores exceeded 40 mm, requiring rescue analgesia. A statistically significant difference was observed in terms of changes in rest VAS scores (*p* = 0.015). Significant differences were also observed in the changes in cough VAS scores over time (*p* = 0.003). Nausea and vomiting were observed in 2 patients (10%). No other complications were observed during the follow-up period.

**Conclusions:**

Multimodal analgesia, including CSAPB, appears to be a feasible regional analgesia technique associated with low postoperative pain scores and minimal rescue analgesic requirements in patients undergoing MICS via right mini-thoracotomy.

**Trial registration:**

Clinicaltrials Registration No: NCT06326320, Registration Date: 17/03/2024.

## Background

Recent advances in percutaneous coronary intervention (PCI) techniques have prompted cardiovascular surgeons to re-evaluate the invasive nature of traditional surgical revascularization procedures, leading to the development of minimally invasive cardiac surgery (MICS) approaches [1]. The primary objective of MICS procedures is to minimize postoperative morbidity by obviating the necessity for full sternotomy [2]. The extant literature demonstrates that MICS procedures result in a number of benefits, including reduced blood product transfusion requirements, shorter intubation times, significant reductions in intensive care and hospital stay durations, decreased postoperative pain intensity, and faster return to daily activities [2–4].

MICS techniques performed via right thoracic mini-thoracotomy are widely utilized in various cardiac procedures, including mitral valve replacement (MVR), aortic valve replacement (AVR), and minimally invasive direct coronary artery bypass grafting (MIDCAB) [5]. However, MICS procedures performed via mini-thoracotomy are not without technical challenges and inherent risks due to difficulties in achieving adequate surgical access, a limited visual field, and the presence of complex anatomical variations. Inadequate surgical visual field has been demonstrated to result in protracted operation times and an elevated incidence of perioperative complications. Aggressive retraction of the intercostal spaces and traction of the intercostal nerves can result in significant pain in the postoperative period [6–8].

Intravenous opioids have historically constituted the fundamental component of analgesic therapy in cardiac surgery procedures. However, there has been an emergence of multimodal approaches that prioritize reduced opioid use in MICS, with the objective of optimizing the postoperative recovery process [4]. The utilization of regional anesthesia techniques during the preoperative period has been demonstrated to result in a substantial reduction in intraoperative opioid requirements, while contributing to postoperative pain control [9, 10]. In MICS patients, various ultrasound-guided thoracic regional anesthesia techniques are utilized, including thoracic paravertebral block (TPVB), erector spinae plane block (ESPB), deep and superficial serratus anterior plane blocks (D-S SAPB), and pectoral nerve blocks (PECS I-II). These blocks have become compatible with Enhanced Recovery After Surgery (ERAS) protocols due to their potential to reduce opioid use and facilitate postoperative mobilization [6, 11, 12]. The rationale for favoring these techniques is multifaceted, encompassing factors such as their ease of application under ultrasound guidance, reliance on anatomical landmarks, relatively low procedural risks, and minimal equipment requirements [8, 13, 14].

SAPB is an ultrasound-guided fascial plane block technique used in the treatment of thoracic wall pain, first described by Blanco [15]. This technique targets the lateral branches of the thoracic intercostal nerves in the lateral thoracic wall and is typically applied at the 4th–6th intercostal levels [15]. The SAPB procedure entails the injection of local anesthetic into either the superficial layer (between the serratus anterior and latissimus dorsi muscles) or the deep layer (between the serratus anterior and ribs) of the serratus anterior muscle [11, 16]. Combined SAPB (CSAPB) techniques, involving simultaneous injection into both superficial and deep planes, have been proposed to enhance block efficacy [17–19]. This approach ensures that the local anesthetic spreads more widely along the thoracodorsal and lateral thoracic nerves, enabling multiple segments of the intercostal nerves to be affected. Multi-plane injection techniques have also been demonstrated to mitigate the risk of block failure when compared with single-plane block applications [8, 10]. In addition, multi-plane injection techniques have been shown to be more effective in reducing opioid requirements [20, 21].

Postoperative pain intensity may be high in MICS patients due to the effect of lateral thoracotomy on intercostal nerves [8, 15]. The present study therefore aimed to evaluate the feasibility and postoperative analgesic profile associated with intraoperative CSAPB, as well as its relationship with opioid requirements, in patients undergoing MICS. Multimodal analgesia including CSAPB was hypothesized to be associated with reduced postoperative pain levels.

## Materials and methods

### Study design and patients

The study was conducted using a prospective, observational feasibility design following approval from the Ankara Bilkent City Hospital Ethics Committee (E.Kurul-E2-24-6175, 07/02/2024). Written informed consent was obtained from all participants included in the study. The trial was registered on ClinicalTrials.gov (Trial registration number: NCT06326320; date: 17/03/2024; principal investigator: Emine Nilgün Zengin, MD), and patient enrollment began after registration.

Patients aged 18 to 80 years with an American Society of Anesthesiologists (ASA) physical status classification of II or III and a body mass index (BMI) between 18 and 40 kg/m² undergoing MICS were included in the study. Exclusion criteria were: age under 18 or over 80 years, American Society of Anesthesiologists (ASA) physical status classification of I, IV or higher, BMI below 18 kg/m² or above 40 kg/m², presence of infection at the block site, preoperative acute or chronic pain, history of opioid therapy, patients not undergoing MICS, and surgeries initially planned as MICS but converted to open procedures. Preoperative anesthesia assessments were completed for 20 patients who provided written informed consent by signing the informed consent form.

#### Interventions

All patients were monitored using standard cardiac anesthesia monitoring, including electrocardiography (ECG), pulse oximetry, capnography, invasive arterial blood pressure, central venous pressure, intraoperative transesophageal echocardiography (TEE), processed electroencephalography (Bispectral index (BIS), Aspect) monitoring, near-infrared spectroscopy (INVOS, NIRS), and temperature monitoring. Moreover, external defibrillator pads were positioned appropriately before draping to enable immediate intervention in the event of arrhythmias or fibrillation. Anesthesia induction included propofol (1.5 mg·kg^− 1^), fentanyl (1–2 µg·kg^− 1^), and rocuronium (1 mg·kg^− 1^) after preoxygenation. The intubation process was performed using an 8.0–8.5 mm single-lumen endotracheal tube, with the objective of minimising the risk of reintubation and ensuring patient comfort. This approach was adopted to facilitate extubation in the postoperative intensive care unit (ICU). A TAPPA bronchial blocker was placed in the right main bronchus under guidance, and its placement was confirmed by fibreoptic bronchoscopy (FOB). Lung isolation was achieved using the TAPPA blocker to improve access to and visualisation of the heart and major vessels, and one-lung ventilation (OLV) was used in all patients. Anesthesia was maintained with desflurane in an oxygen/air mixture in combination with remifentanil. The BIS was maintained between 40 and 50. Remifentanil was administered as a continuous infusion at a rate of 0.05 to 0.25 µg·kg^− 1^·min^− 1^ titrated based on alpha wave patterns observed on the BIS density spectral array (DSA). As a component of multimodal analgesia, nerve blocks were performed under general anesthesia, with the patient in the supine position, and were guided by ultrasound prior to the start of the surgical procedure.

##### C*SAPB*

**Fig. 1:**
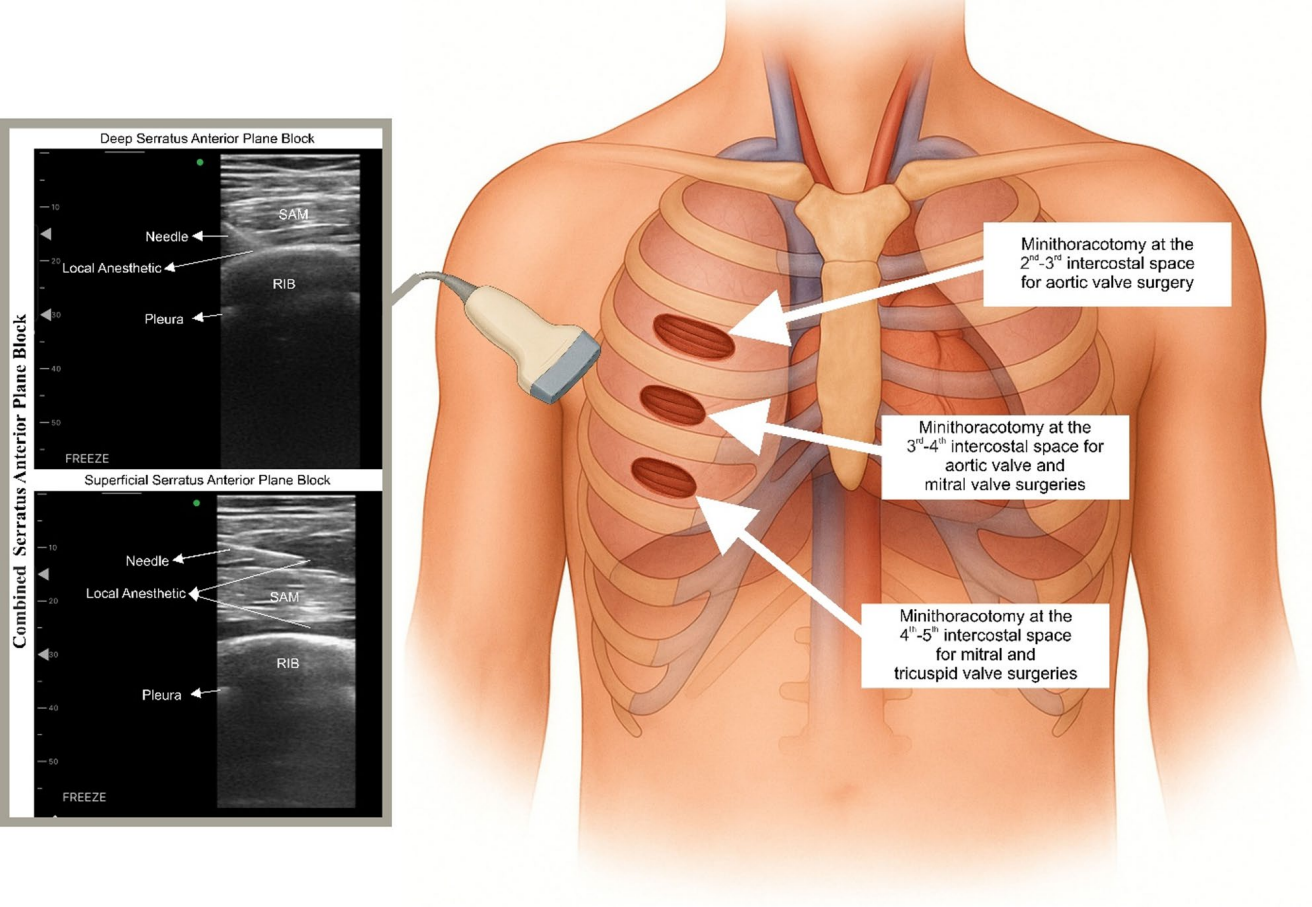
Illustrative image of block and surgical procedures (SAM: serratus anterior muscle)

As part of a multimodal analgesia strategy, ultrasound-guided CSAPB was performed after induction of general anesthesia and prior to surgical incision, as a preemptive analgesic intervention. An ultrasound-compatible 22-gauge, 80-mm nerve block needle and a high-frequency (6–18 MHz) linear probe were used for block application. Standard aseptic precautions were strictly followed. The skin over the lateral thoracic wall was disinfected using an appropriate antiseptic solution, and a sterile ultrasound probe cover and sterile gel were applied. The linear ultrasound probe was positioned in the mid-axillary line at the level of the fourth rib. Under ultrasound guidance, the relevant anatomical structures were systematically identified, including the latissimus dorsi muscle superficially, the serratus anterior muscle beneath it, the underlying ribs, and the pleural line. Care was taken to clearly visualize the pleura as a hyperechoic sliding structure to avoid inadvertent puncture. A block needle was introduced using an in-plane technique from a cranial-to-caudal direction, allowing continuous visualization of the needle shaft and tip throughout the procedure. The needle was advanced carefully until contact with the fourth rib was achieved, ensuring accurate positioning within the deep serratus anterior plane (between the serratus anterior muscle and the rib surface). After negative aspiration, hydrodissection was performed with 2 mL of normal saline to confirm correct needle placement and to open the interfascial plane. Subsequently, 20 mL of 0.25% bupivacaine was injected incrementally into the deep serratus anterior plane, with real-time ultrasound visualization of local anesthetic spread along the rib surface. Following completion of the deep plane injection, the needle was withdrawn approximately 1–2 cm to reposition the tip into the superficial serratus anterior plane (between the serratus anterior and latissimus dorsi muscles). Correct positioning was again confirmed using hydrodissection with 2 mL of normal saline. A further 20 mL of 0.25% bupivacaine was then administered incrementally into the superficial plane, ensuring adequate longitudinal spread of the local anesthetic. Throughout the procedure, intermittent aspiration was performed to minimize the risk of intravascular injection. The distribution of the local anesthetic was continuously monitored under ultrasound to ensure effective interfascial spread in both planes. No immediate complications were observed during block application (Fig. 1).

All blocks were performed by the same experienced anesthesiologist. Dermatomal sensory assessment was not performed due to the block being applied under general anesthesia. Following the block applications, the surgical procedure commenced. Depending on the planned procedure, standard surgical procedures included a 4–5 cm right anterior minithoracotomy (RAT) at the 2nd -3rd intercostal space for aortic valve surgery, and a 4–5 cm RAT at the 3rd -4th intercostal space for aortic valve and mitral valve surgery. Finally, a 4–5 cm RAT was performed at the 4th -5th intercostal space for mitral and tricuspid valve surgery (Fig. 1).

At the end of the surgery, a chest tube was inserted three intercostal spaces below the level of the surgical incision. The bronchial blocker was removed. Prior to transfer to the intensive care unit (ICU), all patients received 1 gram of intravenous acetaminophen and 1 mg·kg^− 1^ of intravenous tramadol. Subsequently, patients were transferred to the ICU while still intubated. In the ICU, patients were sedated with remifentanil and supported with mechanical ventilation. Once hemodynamic stability and normothermia were achieved, sedation was discontinued, patients were extubated, and mechanical ventilation was weaned. Patients received four doses of 1 gram of acetaminophen within the first 24 h in the ICU. Visual Analog Scale (VAS) pain scores were recorded at the 0th (after extubation with an Aldrete score ≥ 9), 2nd, 4th, 8th, and 12th hours following extubation. Rescue analgesia was administered based on the reported pain scores. If a patient’s VAS score exceeded 40 mm at any time, the initial treatment consisted of tramadol (1 mg·kg^− 1^). If pain persisted, morphine (0.5 mg·kg^− 1^) was administered as needed.

A comprehensive patient demographics database was collated, encompassing patient age, gender, and BMI. In addition, operative details such as intraoperative remifentanil consumption, surgical duration, and time to extubation were documented. Time to extubation was defined as the interval from the end of surgery to successful tracheal extubation. Any major complications occurring during block application or postoperative follow-up, including bleeding, nausea, vomiting, bradycardia, tachycardia, hypotension, and delirium, were documented. Postoperative VAS scores, the need for additional analgesics, and monitoring parameters such as mean blood pressure (MBP), heart rate (HR), and oxygen saturation (SpO₂) were also documented.

## Outcome measurements

The primary outcome was postoperative pain intensity, assessed using the VAS at predefined time points during the first 24 postoperative hours. Secondary outcomes included intraoperative remifentanil consumption, total postoperative tramadol consumption, rescue analgesia requirements, hemodynamic parameters, duration of postoperative mechanical ventilation, length of ICU stay and hospital stay, and patient satisfaction.

### Statistical analysis

All collected data were analyzed utilizing the Jamovi statistical program version 2.3.21.0 (Sydney, Australia). The Shapiro-Wilk test was employed to evaluate normality of the data distribution. Normally distributed data were expressed as mean ± standard deviation (SD), while non-normally distributed or ordinal data were presented as median and interquartile range (IQR). Categorical variables were represented as counts (n) and percentages (%). Univariate repeated measures were adjusted according to the result of the analysis of variance (ANOVA), and epsilon (ε) values were calculated according to Greenhouse-Geisser. Significance levels for multiple tests were adjusted using the Bonferroni correction. Repeated measurements of continuous variables, including VAS scores (at rest and during coughing), heart rate, and mean blood pressure, were compared across predefined postoperative time points (0, 2, 4, 8, and 12 h) using repeated measures analysis of variance (ANOVA). Greenhouse–Geisser correction was applied when the assumption of sphericity was violated. Post hoc pairwise comparisons were performed using Bonferroni correction to identify differences between time points. Thus, all statistical analyses were based on within-subject (intra-individual) comparisons over time. Given the small sample size, all statistical analyses were considered exploratory and intended to describe within-subject temporal trends rather than to provide confirmatory inference. Statistical significance was determined for p values less than 0.05.

## Results

A total of 20 patients who underwent minimally invasive cardiac surgery were included in the study. Mitral valve replacement was performed in 10 patients (50%), aortic valve replacement in 3 patients (15%), and tricuspid valve replacement in 1 patient (5%). Combined mitral and tricuspid valve replacement was performed in 4 patients (20%), while 2 patients (10%) underwent combined mitral and aortic valve replacement. The mean age of the patients was 61 years. Of the patients, 8 (40%) were female and 12 (60%) were male. The mean BMI was 27.7 kg/m². Detailed demographic data of the patients are presented in Table 1.

**Table 1:** Demographic data of the patients

Case No	Age (Year)	Gender (F/M)	ASA	BMI kg/m^2^	Comorbidity	Procedure
1	54	M	2	29.75	-	MVR
2	74	F	3	25.91	HT, Asthma	MVR
3	53	F	3	34.92	HT, AF	MVR
4	65	F	3	30.35	AF	MVR
5	60	F	3	34.62	HT	MVR
6	54	F	3	28.95	DM, Asthma, Hypothyroidism	MVR, AVR
7	58	M	2	25.7	-	MVR
8	74	M	3	29.41	HT, AF	MVR, AVR
9	71	M	3	24.4	HT, AF	MVR, TVR
10	52	M	3	19.97	HT, AF	MVR, TVR
11	74	M	3	30.44	CAD, DM	AVR
12	52	F	3	25.1	COPD	MVR
13	52	F	2	24.5	-	MVR, TVR
14	59	M	3	22.03	HT	MVR
15	54	M	2	31.2	-	MVR
16	63	M	3	22.53	AF	TVR
17	65	M	3	31.51	HT, DM	AVR
18	57	M	2	24.61	-	MVR
19	64	M	3	28.54	HT, CVD, Epilepsy	AVR
20	64	F	3	28.88	AF, DM, CVD	MVR, TVR
Mean	61.0 ± 7.76	8/12	-	27.7 ± 4.07	-	-
Median (IQR)	59.5 (11)	-	-	28.7 (5.79)	-	**-**

ASA: American Society of Anesthesiologists; AF: atrial fibrillation; AVR: aortic valve replacement; BMI: body mass index; CAD: coronary artery disease; COPD: chronic obstructive pulmonary disease; CVD: cerebrovascular disease; DM: Diabetes mellitus; F: Female; HT: Hypertension; M: Male; MVR: mitral valve replacement; TVR: tricuspid valve replacement

The mean surgical duration was 256 min, while the average cardiopulmonary bypass (CPB) time was 154 min. The mean duration of anesthesia was 301 min. Intraoperatively, patients consumed a mean total remifentanil of 2350 ± 450 µg, corresponding to 31.5 ± 6.2 µg·kg⁻¹ total dose and a mean infusion rate of 0.105 ± 0.016 µg·kg⁻¹·min⁻¹. Patients were extubated approximately 310 min after the operation. The average length of ICU stay was 1 day, and the mean hospital stay was 5 days. Procedural and follow-up data are detailed in Table 2.

**Table 2:** Procedure and follow-up data

Case No	Operation duration; (min)	Pump duration; (min)	Anesthesia duration; (min)	Remifentanil requirement (µg)	Extubation time; (min)	Length of intensive care; (day)	Length of hospital; (day)
1	210	122	255	2100	300	1	6
2	220	150	260	1800	330	2	7
3	320	161	340	2600	325	1	5
4	196	111	273	1700	320	2	5
5	270	194	300	2200	325	2	6
6	280	185	330	2500	300	2	6
7	300	180	340	2800	280	1	6
8	306	225	370	3400	340	1	7
9	330	230	375	3200	270	1	7
10	257	158	315	2300	315	2	7
11	193	94	250	1900	320	1	6
12	255	115	290	2200	280	1	5
13	285	165	315	2400	310	1	5
14	209	122	258	1800	330	1	4
15	250	180	285	2600	280	1	5
16	145	85	190	2100	300	2	7
17	230	130	270	2000	305	1	5
18	240	196	300	2100	330	1	5
19	320	182	360	2800	300	1	4
20	310	90	350	2400	340	2	6
Mean	256 ± 50.8	154 ± 43.5	301 ± 47.6	2350 ± 457	310 ± 21	1.35 ± 0.48	5.70 ± 0.97
Median (IQR)	256 (84)	160 (62.5)	300 (72.5)	2250 (525)	313 (26.3)	1 (1)	6 (1.25)

**Fig. 2:**
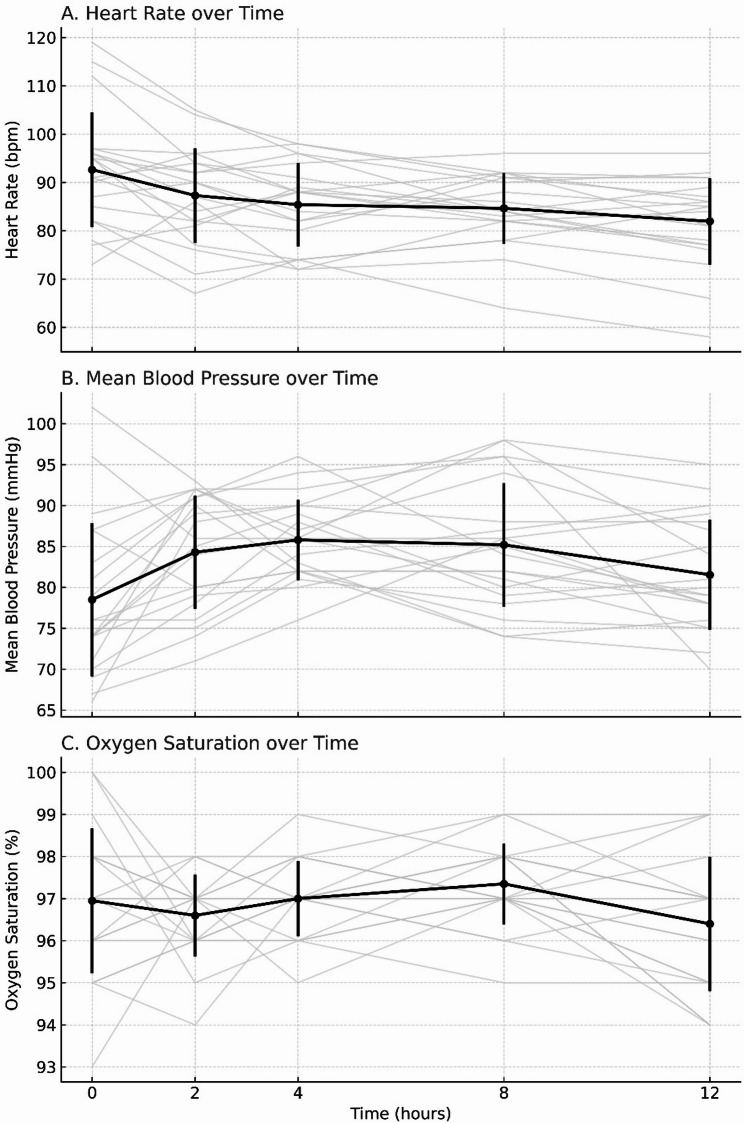
Individual patient trends and group mean ± SD for heart rate (**A**), mean blood pressure (**B**), and oxygen saturation (**C**) at 0, 2, 4, 8, and 12 h postoperatively. (Gray lines represent individual patient values (*n* = 20), and the black line with error bars indicates the mean ± standard deviation at each time point.)

The hemodynamic data for each patient during the post-extubation follow-up period are presented in Fig. 2. Statistically significant differences were determined in terms of HR (p = < 0.001, Greenhouse-Geisser, ε = 0.653) and MBP (*p* = 0.015, Greenhouse-Geisser, ε = 0.566) at the time points monitored in hemodynamic data. HR was significantly lower at the 4th, 8th, and 12th hour time points in comparison to the 0th hour time point. The significant difference in MBP was attributable to the difference between the 4th hour time point and the 0th hour time point. Hemodynamic parameters remained within clinically acceptable ranges throughout the perioperative period.

At rest, VAS scores were assessed at the 0th, 2nd, 4th, 8th, and 12th hours after extubation (Aldrete score ≥ 9). In 2 patients (10%), VAS scores exceeded 40 mm, and rescue analgesia was successfully achieved with tramadol alone (a total of 145 mg of tramadol was administered to the two patients), with no requirement for morphine in any patient. In one of these patients, the VAS score was above 40 mm at the 0th hour, and in the other, at the 8th hour. The patient reporting high resting pain at the 0th hour also had a cough VAS score exceeding 60 mm. When changes in rest VAS scores were analyzed over time, a statistically significant difference was observed (*p* = 0.015, Greenhouse-Geisser, ε = 0.688). The VAS rest value at the 4th hour time point was the lowest, and there was a statistically significant difference of 6.4 mm on average compared to the 0th hour time point. Significant differences were also observed in the changes in cough VAS scores over time (*p* = 0.003, Greenhouse-Geisser, ε = 0.718). The reason for the difference in cough VAS scores across time intervals was an average decrease of 5.15 mm in the 2nd hour time interval compared to the 0th hour time interval and a statistically significant decrease of 7.05 mm in the 4th hour time interval.

**Fig. 3:**
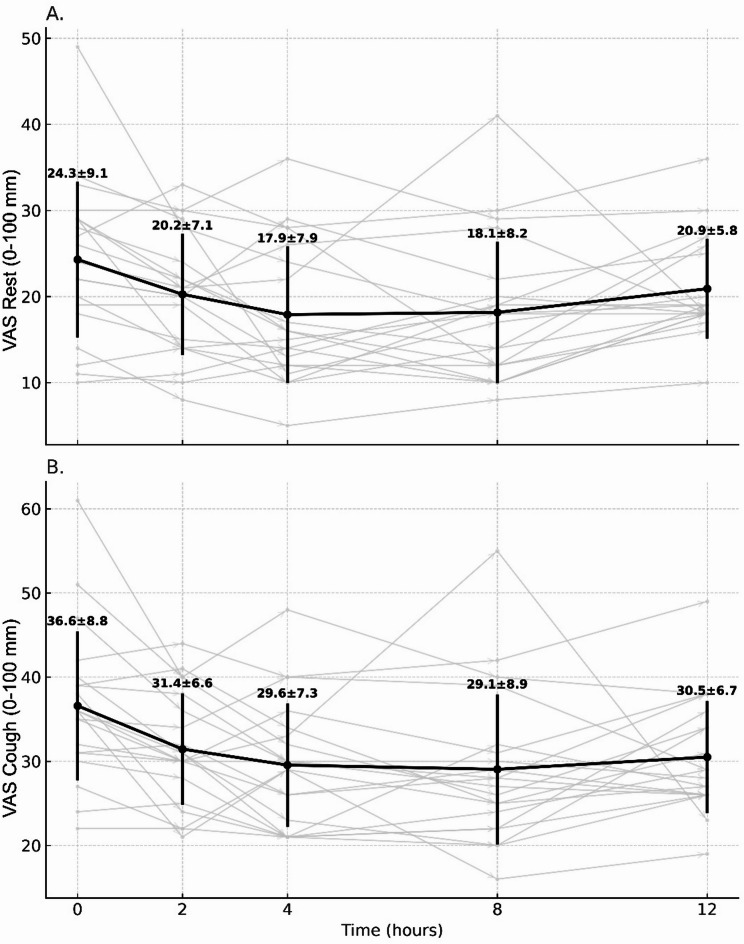
Pain scores at rest (**A**) and during coughing (**B**) measured at 0, 2, 4, 8, and 12 h postoperatively. (Gray lines with arrows represent individual patient trajectories (*n* = 20), and the black line with error bars indicates the mean ± standard deviation.)

The mean resting VAS pain scores at each time point were as follows (Fig. 3):

– 24.3 ± 9.1 mm at the 0th hour.
– 20.2 ± 7.1 mm at the 2nd hour.
– 17.9 ± 7.9 mm at the 4th hour.
– 18.1 ± 8.2 mm at the 8th hour.
– 20.9 ± 5.8 mm at the 12th hour.

The mean cough VAS scores were:

– 36.6 ± 8.8 mm at the 0th hour.
– 31.4 ± 6.6 mm at the 2nd hour.
– 29.6 ± 7.3 mm at the 4th hour.
– 29.1 ± 8.9 mm at the 8th hour.
– 30.5 ± 6.7 mm at the 12th hour.

**Fig. 4:**
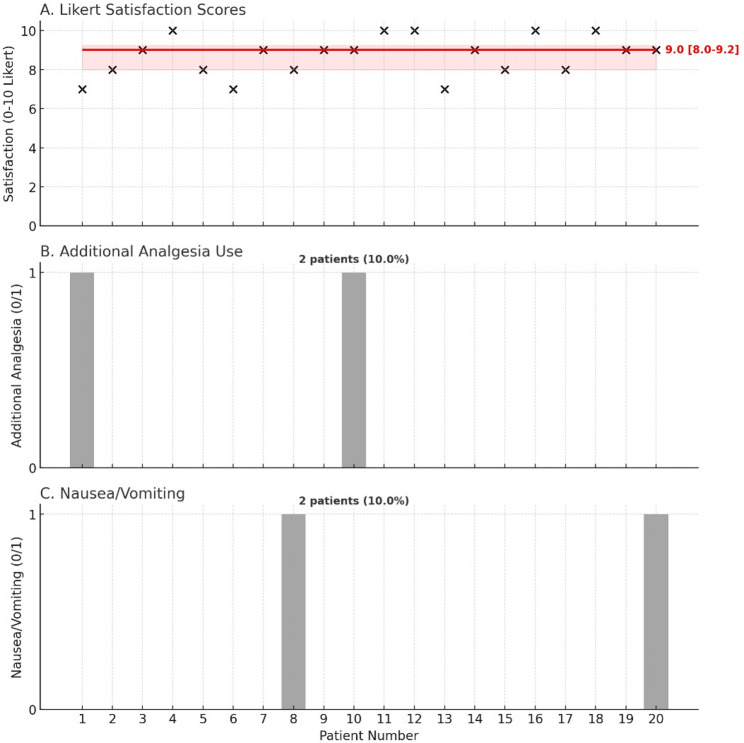
Patient-reported satisfaction scores (**A**), additional analgesic requirement (**B**), and incidence of nausea and vomiting (**C**) during the postoperative period. (In panel A, black dots represent individual patient satisfaction scores on a 0–10 Likert scale, the red horizontal line denotes the median, and the shaded area shows the interquartile range. In panels B and C, bars indicate the presence (1) or absence (0) of additional analgesic use and nausea and vomiting.)

During the follow-up period, postoperative nausea and vomiting (PONV) was observed in 2 patients (10%). These two patients also had the longest extubation times. No cases of respiratory depression were detected. No other complications were observed during the follow-up period. The median Likert satisfaction score among patients was 9. Patient-specific satisfaction scores and observed adverse events are detailed in Fig. 4.

## Discussion

Prospective data on the analgesic profile of CSAPB as part of multimodal analgesia in patients undergoing MICS via right mini-thoracotomy are scarce, and intraoperative opioid consumption within CSAPB-based protocols in this surgical context has not previously been reported in normalised units. In this observational feasibility study, the combination of preemptive CSAPB with scheduled acetaminophen was associated with consistently low postoperative pain scores throughout the 12-hour follow-up period, a low rescue analgesia requirement, an intraoperative opioid profile within the range reported for regional analgesia-based MICS protocols, and high patient satisfaction. These findings position CSAPB as a feasible component of multimodal analgesia in MICS and provide preliminary data to inform the design of future randomised controlled trials. Given the absence of a control group, causal inference cannot be established; the results should be interpreted as descriptive and hypothesis-generating.

MICS has been increasingly adopted over the past two decades due to its association with reduced surgical trauma, decreased need for transfusion, shorter ICU and hospital stays, and accelerated postoperative recovery [2–4]. Despite these advantages, optimal postoperative pain control remains a significant challenge, particularly in thoracotomy-based MICS procedures, which involve intercostal nerve manipulation and rib retraction, both of which contribute to severe somatic and visceral pain [11, 12].

The SAPB targets the lateral cutaneous branches of the intercostal nerves within the serratus anterior fascial plane, providing dermatomal coverage from T2 to T9, a distribution that encompasses both the surgical incision and the chest tube insertion site in right mini-thoracotomy MICS [15]. Fascial plane blocks deliver analgesia through bulk flow and diffusion of local anesthetic within interfascial planes, with onset, dermatomal coverage, and duration governed by fascial microanatomy, collagen architecture, and individual anatomical variation rather than by direct bathing of discrete nerve trunks [22]. This mechanism underlies the inherent variability in block onset and duration that distinguishes fascial plane blocks from neuraxial or peripheral nerve block techniques, and must be kept in mind when interpreting postoperative pain trajectories. In the most directly comparable published series, Marianello et al. [7] reported that postoperative deep SAPB in 50 patients undergoing MVR via right anterior mini-thoracotomy reduced rescue opioid requirements from 84% to 20% (*p*<0.001), shortened mechanical ventilation from 14 to 4 h, and reduced ICU stay from 2 to 1 day. Our rescue analgesia rate of 10%, extubation time of 310 min, and 1-day ICU stay are consistent with this SAPB-treated cohort, despite our use of preemptive CSAPB rather than postoperative deep SAPB. In a prospective randomised trial comparing CSAPB and deep SAPB in VATS, Zengin et al. [19] reported resting VAS of approximately 30 mm at the 1st hour declining to 10 mm at 24 h in the CSAPB group, with a rescue analgesia rate of 23.3%. Our resting VAS of 24.3 ± 9.1 mm at extubation, declining to 17.9 ± 7.9 mm at the 4th hour and remaining stable at 20.9 ± 5.8 mm at the 12th hour, reflects a comparable analgesic trajectory in a surgically more demanding cardiac setting. The within-subject reductions of 6.4 mm at rest and 7.05 mm during coughing fall below the conventional minimal clinically important difference (MCID) of approximately 13 mm for acute postoperative pain [23, 24]; however, mean baseline VAS at extubation was already within the mild pain range, inherently constraining the achievable reduction. More clinically informative is the temporal stability: VAS scores did not exceed baseline values at any point across the 12-hour window, no patient required escalation beyond tramadol, and the median satisfaction score was 9 out of 10. Given the variable and prolonged nature of fascial plane block analgesia, this sustained absence of pain escalation over 12 h reflects the analgesic continuity provided by the multimodal protocol, and constitutes the primary indicator of analgesic stability in this cohort.

There are several factors that influence the success of fascial plane blocks, one of which is the presence of septa within the fascial layers [8]. In recent years, dual injections have been increasingly employed to enhance local anesthetic spread and improve block efficacy with results suggesting potential analgesic benefit [11, 16, 17]. Performing a dual-level injection (both superficial and deep to the serratus anterior muscle) appears to increase the spread of local anesthetic across interfacial planes, potentially minimizing the risk of block failure due to anatomical barriers such as fascial septa. This technique may also contribute to the more extensive blockade of the lateral cutaneous branches of the intercostal nerves, long thoracic nerve, and thoracodorsal nerve, which may otherwise be inconsistently affected in single-injection approaches. For example, Zengin et al. [11] used a volume of 10 + 10 mL for CSAPB administration in patients undergoing VATS, whereas Ülger et al. [17] utilized a volume of 15 + 15 mL in similar cases. In parallel with this, Şimşek et al. [25] performed the CSAPB using a total volume of 30 mL (15 + 15 mL) of 0.375% bupivacaine with epinephrine (5 µg/mL). In the same study, an additional 5 mL of 0.25% bupivacaine was administered for the chest tube, resulting in a total volume of 35 mL. Özgüner et al. [16] employed a 15 + 15 mL volume for CSAPB in the postoperative analgesia of breast surgery and reported adequate analgesic outcomes. Blanco used a volume of 4 mL·kg^− 1^ (19–29 mL) for a single fascial plane block [15]. To our knowledge, prospective data on CSAPB specifically in MICS remain scarce; we therefore opted for a relatively higher volume (20 + 20 mL) to ensure sufficient dermatomal coverage. In this context, considering that the spread and efficacy of fascial plane blocks are highly volume-dependent and influenced by multiple anatomical and technical factors, our preference for a higher total injectate volume was intended to maximize longitudinal spread and dermatomal coverage while maintaining an acceptable safety profile, as the total administered dose of bupivacaine remained within established safety limits (< 2.5 mg/kg) [8, 26]. As the block was performed unilaterally in the setting of single-sided surgery, cumulative local anesthetic exposure was limited, and the risk of local anesthetic systemic toxicity (LAST) was therefore considered to be low.

Intraoperative opioid management in MICS is increasingly guided by the principle of opioid economy, with regional analgesia used to reduce reliance on high-dose remifentanil and its associated risks of delayed weaning and postoperative respiratory depression [4, 13]. Prospective data on intraoperative opioid consumption specifically within CSAPB-based protocols for MICS are absent from the published literature. Baran et al. [10], in the only available randomised trial of preemptive fascial plane blocks in MICS, reported intraoperative remifentanil consumption of 2468.0 µg (1803.0–3018.0) in the ESPB group and 2382.0 µg (1814.0–3199.0) in the IPB+SAPB group, at a fixed infusion rate of 0.1 µg·kg⁻¹·min⁻¹ in procedures of comparable duration. In our cohort, mean total intraoperative remifentanil consumption was 2350 ± 450 µg, corresponding to 31.5 ± 6.2 µg·kg⁻¹ total dose and a mean infusion rate of 0.105 ± 0.016 µg·kg⁻¹·min⁻¹. This rate falls within the range reported by Baran et al. [10] for alternative fascial plane block techniques in MICS, and aligns with the infusion range of 0.05–0.1 µg·kg⁻¹·min⁻¹ reported by Marianello et al. [7] in the MVR via RAT setting. These findings suggest that CSAPB, as part of a multimodal analgesia regimen, is associated with an intraoperative opioid profile consistent with published MICS series using regional analgesia, though the absence of a control group precludes attribution of opioid reduction to the block specifically.

A clinically relevant characteristic of SAPB is its lack of sympathetic or central neuraxial involvement. Unlike intrathecal morphine, thoracic epidural block or TPVB, SAPB does not induce significant sympathetic blockade, which minimizes the risk of hemodynamic instability [7, 27]. Our hemodynamic observations are consistent with this, as no patient experienced clinically significant hypotension or bradycardia attributable to the block. Although minor variations in heart rate and mean blood pressure were observed, these changes likely reflect normal perioperative physiological responses rather than a direct effect of the block. In a study by Richa Dhawan et al. [9], nausea was reported in 12 patients (36.4%) following intrathecal morphine administration. Marianello et al. [7] compared postoperative SAPB and opioid groups and found the incidence of delirium to be 3 (6%) and 12 (22%), respectively. The incidence of PONV was reported as 9 (18%) in the SAPB group and 6 (11%) in the opioid group. In our study, a lower incidence of PONV (2 patients, 10%) was observed, which may be attributed to reduced opioid consumption and the avoidance of central neuraxial techniques. No other complications related to the block, such as respiratory depression and bleeding, were observed, which reinforces the safety profile of this technique in the cardiac surgery population.

Supine positioning represents a practical advantage of CSAPB over alternative regional techniques in the cardiac surgery setting [28]. In contrast, TPVB, ESPB, or newer techniques such as the sub-pleural intercostal plane block typically require lateral decubitus positioning and are either difficult or not feasible to perform intraoperatively in anesthetized patients. Preoperative administration of these blocks in the awake patient may lead to increased anxiety and discomfort. In our protocol, CSAPB was administered without complication under general anesthesia with the patient in the supine position, avoiding such concerns.

This study has some limitations. Due to the absence of a control group, causal inference regarding the analgesic efficacy of CSAPB cannot be established; therefore, the findings should be interpreted as descriptive and hypothesis-generating. First, the relatively small number of patients (*n* = 20), together with the absence of a priori power calculation, limits the generalizability and statistical robustness of the findings. Therefore, the results of the statistical analyses should be interpreted as exploratory. Second, the absence of a comparator group prevented direct assessment of CSAPB against alternative techniques such as TPVB or systemic analgesia alone. In addition, all patients received a standardized multimodal analgesia regimen, including acetaminophen and tramadol, which may confound the ability to isolate the specific contribution of CSAPB to postoperative analgesia. Therefore, the findings should be interpreted as reflecting the combined effect of multimodal analgesia including CSAPB. Third, the follow-up period was limited to the early postoperative phase (12 h), and long-term pain outcomes, including chronic post-thoracotomy pain, were not assessed. Fourth, there was a lack of patient-controlled analgesia; this prevented accurate measurement of total opioid consumption and the time to first analgesic request. However, the low requirement for rescue analgesia and the absence of morphine use suggest feasible analgesia. Fifth, this study did not include standardized measurements of functional recovery parameters such as mobilization and respiratory function, or long-term pain outcomes. Therefore, the broader clinical impact of CSAPB within enhanced recovery protocols could not be fully evaluated. Finally, the lack of objective quantification of dermatomal spread with sensory testing may limit the precision of our block effectiveness evaluation.

In conclusion, CSAPB appears to be a safe and feasible regional analgesia technique in patients undergoing MICS via RAT. In this observational study, its use was associated with low postoperative pain scores and minimal rescue analgesic requirements, while hemodynamic parameters remained within clinically acceptable ranges. However, due to the absence of a control group, causal relationships cannot be established. Further randomized controlled trials with larger sample sizes are warranted to confirm these findings and to determine the optimal local anesthetic volume and concentration for CSAPB in the MICS population.

## Data Availability

The datasets used and/or analysed during the current study available from the corresponding author on reasonable request.

## Abbreviations

ASA: American Society of Anesthesiologists
AVR: Aortic valve replacement
BIS: Bispectral index
BMI: Body mass index
CPB: Cardiopulmonary bypass
CSAPB: Combined serratus anterior plane block
DSA: Density spectral array
D-S SAPB: Deep and superficial serratus anterior plane blocks
ECG: Electrocardiography
ERAS: Enhanced Recovery After Surgery
ESPB: Erector spinae plane block
FOB: Fibreoptic bronchoscopy
HR: Heart rate
ICF: Informed consent form
ICU: Intensive care unit
IPB: Interpectoral plane block
IQR: Interquartile range
MBP: Mean blood pressure
MICS: Minimally invasive cardiac surgery
MIDCAB: Minimally invasive direct coronary artery bypass grafting
MVR: Mitral valve replacement
NIRS: Near-infrared spectroscopy
OLV: One-lung ventilation
PCI: Percutaneous coronary intervention
PECS: Pectoral nerve blocks
RAT: Right anterior minithoracotomy
SAPB: Serratus anterior plane block
SD: Standard deviation
SpO_2_: Oxygen saturation
TEE: Transesophageal echocardiography
TPVB: Thoracic paravertebral block
VAS: Visual analog scale
